# Stroke–heart syndrome and risk of incident dementia among patients with first‐ever ischemic stroke: A territory‐wide population‐based cohort study

**DOI:** 10.1002/alz.70716

**Published:** 2025-09-18

**Authors:** Christopher T. W. Tsang, Sylvia E. Choi, Tommaso Bucci, Alfred C. W. Lau, Qing‐Wen Ren, Jia‐Yi Huang, Mei‐Zhen Wu, Wen‐Li Gu, Ran Guo, Jing‐Nan Zhang, Yuen‐Ching Ng, Benjamin J. R. Buckley, Jan F. Scheitz, Yap‐Hang Chan, Kui‐Kai Lau, Hung‐Fat Tse, Azmil H. Abdul‐Rahim, Gregory Y. H. Lip, Kai‐Hang Yiu

**Affiliations:** ^1^ Division of Cardiology The University of Hong Kong Shen Zhen Hospital Shenzhen China; ^2^ Cardiology Division Department of Medicine The University of Hong Kong Hong Kong China; ^3^ Liverpool Centre for Cardiovascular Science at University of Liverpool Liverpool John Moores University and Liverpool Heart & Chest Hospital Liverpool UK; ^4^ Department of Cardiovascular and Metabolic Medicine Institute of Life Course and Medical Sciences University of Liverpool Liverpool UK; ^5^ Department of Clinical Internal Anesthesiological and Cardiovascular Sciences Sapienza University of Rome Rome Italy; ^6^ Department of Geriatrics National Key Clinical Specialty Guangzhou First People's Hospital School of Medicine South China University of Technology Guangzhou China; ^7^ Cardiovascular Health Sciences Research Institute for Sport and Exercise Sciences Liverpool John Moores University Liverpool UK; ^8^ Department of Neurology and Center for Stroke Research Berlin (CSB) Charité‐Universitätsmedizin Berlin Berlin Germany; ^9^ Neurology Division Department of Medicine The University of Hong Kong Hong Kong China; ^10^ State Key Laboratory of Brain and Cognitive Sciences The University of Hong Kong Hong Kong China; ^11^ Stroke Division Department of Medicine for Older People Whiston Hospital Mersey and West Lancashire Teaching Hospitals NHS Trust Prescot UK; ^12^ Danish Center for Health Services Research, Department of Clinical Medicine Aalborg University Aalborg Denmark; ^13^ Department of Cardiology, Lipidology and Internal Medicine with Intensive Coronary Care Unit Medical University of Bialystok Bialystok Poland

**Keywords:** ABC pathway, cardiovascular complications, cognitive impairment, dementia, integrated care, ischemic stroke, stroke, stroke–heart syndrome

## Abstract

**INTRODUCTION:**

The risk of dementia in patients with ischemic stroke and early cardiovascular complications (i.e., stroke–heart syndrome [SHS]) remains underexplored.

**METHODS:**

Patients with first‐ever ischemic stroke in Hong Kong between 2005 and 2020 were included. Multivariable Fine–Gray competing risk analysis was performed after 1:1 propensity score matching to evaluate the association between SHS and the risk of dementia.

**RESULTS:**

Of the 130,605 included patients with ischemic stroke, 12,696 (9.7%) patients developed SHS. Patients with SHS had a 19% increased risk of dementia compared to those without SHS at 1 year post‐stroke, driven mainly by vascular dementia. This increased risk gradually declined and became non‐significant after 3 years post‐stroke. Appropriate antithrombotic therapy and comorbidities optimization were associated with a 32% reduced dementia risk in patients with SHS.

**DISCUSSION:**

SHS is associated with an increased risk of incident dementia. Appropriate antithrombotic therapy and comorbidities optimization post‐stroke may reduce this heightened risk of cognitive impairment.

**Highlights:**

- Association between stroke–heart syndrome (SHS) and dementia was evaluated in a population‐based cohort.
- Development of SHS associated with a 19% increased 1 year risk of dementia.
- The increased risk of dementia gradually declined with each year of follow‐up.
- Integrated post‐stroke management may reduce this heightened risk of cognitive impairment.

## 1 BACKGROUND

Despite improvements in overall stroke survival, medical complications after stroke remain a significant burden that complicates neurological recovery and outcomes in patients with stroke.^1^ In particular, post‐stroke neurocardiogenic injury without the confounding effect of pre‐existing heart disease is under‐recognized. To that end, the term “stroke–heart syndrome” (SHS) was recently introduced to provide a conceptual framework for stroke‐induced cardiovascular complications that arise within the first month after stroke.^2^ SHS has a reported incidence of 10% to 20% among stroke survivors,^3^, ^4^ with those affected presenting with a broad range of manifestations including myocardial injury, acute coronary syndromes, left ventricular dysfunction, arrhythmias, and neurogenic sudden death.^5^ Recent epidemiological studies have shown that patients with SHS were associated with higher risks of recurrent strokes, secondary cardiac events, and death,^6^, ^7^ thus highlighting the need for more intensive monitoring and intervention in this subset of patients.

Apart from cardiovascular outcomes, post‐stroke cognitive impairment and dementia represent another major source of morbidity and mortality after stroke. Stroke survivors are especially vulnerable to cognitive impairment due to the neurovascular damage, cerebral hypoperfusion, and inflammation that follow an ischemic event.^8^ Post‐stroke cognitive impairment significantly impairs patients’ independence, quality of life, and long‐term functional outcomes.^9^ A growing body of evidence shows that both ischemic stroke (IS) and cardiovascular events independently increase the risk of dementia.^10^, ^11^ Despite the established links among cardiovascular disease, stroke, and dementia, the cumulative effect of SHS—a syndrome encompassing stroke and early cardiovascular complications—on dementia risk remains underexplored, especially in Asian populations.

Using a population‐based cohort in Hong Kong, our study aimed to assess the risk of incident dementia in patients with first‐ever IS who developed SHS. Second, we also sought to evaluate the impact of post‐stroke holistic or integrated care management based on appropriate antithrombotic therapy and comorbidities optimization^12^, ^13^ on dementia risk in this cohort, with the hope of informing future post‐stroke care strategies targeting both cardiovascular and neurocognitive health.

## 2 METHODS

### 2.1 Data source

Data in this retrospective cohort study were retrieved from the Clinical Data Analysis and Reporting System (CDARS), an electronic health record database operated by the Hong Kong Hospital Authority. As the sole public health‐care service provider in Hong Kong, CDARS is thus a representative population‐based database that routinely collects data including demographics, diagnoses, procedures, laboratory results, and drug prescriptions. All diagnoses in CDARS are coded by the International Classification of Diseases, Ninth Revision, Clinical Modification (ICD‐9‐CM), which has previously been shown to have good coding accuracy.^14^

This study was conducted in accordance with the Declaration of Helsinki and the Strengthening the Reporting of Observational Studies in Epidemiology (STROBE) statement. As patient data were de‐identified in CDARS, the need for individual consent was waived. The study has been approved by the institutional review board of the University of Hong Kong/ Hospital Authority Hong Kong West Cluster (IRB Reference Number: UW 24‐187).

**RESEARCH IN CONTEXT**

1. **Systematic review**: The authors performed a literature review on the association of stroke–heart syndrome (SHS) and dementia using PubMed. Although previous studies have suggested possible relationships among cardiovascular diseases, stroke, and dementia, the cumulative effect of SHS—a syndrome encompassing stroke and early cardiovascular complications—on dementia risk remains underexplored, especially in Asian populations.
2. **Interpretation**: Development of SHS associated with a 19% increased 1 year risk of incident dementia (especially vascular dementia), which showed a gradual reduction with each successive year of follow‐up and became non‐significant after 3 years post‐stroke. Appropriate antithrombotic therapy and comorbidities optimization post‐stroke were associated with a reduced dementia risk.
3. **Future directions**: Patients with SHS may be at increased risk of dementia in the early phase after a stroke. The utility of standardized post‐stroke protocols, according to the ABC_stroke_ pathway, should be tested in future clinical trials to guide best practices in patient management.

### 2.2 Study population

Patients aged ≥ 18 years with first‐ever IS recorded in CDARS between January 1, 2005 and May 31, 2020 were included. The index date was defined as the date when a patient was diagnosed with IS for the first time.

Based on the development of at least one new‐onset cardiovascular complication, which included heart failure (HF), ischemic heart disease (IHD), atrial fibrillation/atrial flutter (AF/AFL), and ventricular tachycardia/ventricular fibrillation (VT/VF) within 30 days from index stroke, patients were categorized into two groups: patients with SHS and patients without SHS (i.e., patients who experienced IS only). Cardiovascular complications that occurred within 30 days of the index stroke were considered part of the SHS, given the time‐varying risk of major adverse cardiovascular events after IS and the proposed criteria adopted by existing literature.^2^, ^6^, ^15^ To allow the evaluation of incident dementia as our study outcome, patients with pre‐existing Alzheimer's disease (AD), vascular dementia, and unspecified dementia prior to index stroke were excluded. To minimize the confounding effect of heart diseases prior to index stroke and identify patients with new‐onset cardiovascular complications, patients with previous diagnoses of HF, IHD, AF/AFL, VT/VF prior to index stroke, or those who died in the first 30 days after index stroke were also excluded. The flowchart of the study cohort is summarized in Figure S1 in supporting information.

### 2.3 Baseline information

Medical records of each patient were traced back to 5 years prior to the index date until the start of the follow‐up date (i.e., index date + 30 days) for evaluating cohort characteristics. The following data were collected: age, sex, smoking, alcohol use, comorbidities including hypertension (HTN), diabetes mellitus (DM), dyslipidemia, chronic kidney disease (CKD), chronic liver disease (CLD), neoplasms, and medication prescriptions including angiotensin‐converting enzyme inhibitor (ACEi), angiotensin receptor blocker (ARB), beta‐blocker, calcium channel blocker (CCB), diuretics, aspirin, P2Y12 inhibitor, warfarin, non‐vitamin K antagonist oral anticoagulants (NOAC), insulin, metformin, and statin. Baseline medication use was defined by a filled prescription for at least 30 consecutive days prior to the index date. Details of ICD‐9‐CM codes used for data collection are summarized in Table S1 in supporting information.

### 2.4 Study outcomes

Patients were followed for up to 3 years. The primary outcome of this study was the 1 year risk of a composite of AD, vascular dementia, and unspecified dementia. The secondary outcomes were dementia‐related mortality and all‐cause mortality. Details of ICD‐9‐CM codes used to define study outcomes are summarized in Table S1.

### 2.5 Statistical analyses

Continuous variables were reported as mean ± standard deviation, while categorical variables were reported as absolute numbers and percentages. Differences in baseline characteristics between groups were compared using the independent sample *t* test for continuous variables and the chi‐squared test for categorical variables.

Propensity score matching (PSM) at a 1:1 ratio with nearest neighbor matching with a caliper width of 0.01 was used to balance the covariates between patients with and without SHS. With reference to the 2024 report of the Lancet Commission on dementia,^16^ the following variables were included in PSM: age, sex, smoking, alcohol use, baseline comorbidities (HTN, DM, dyslipidemia, CKD, CLD, neoplasms), and baseline medication use (ACEi, ARB, beta‐blocker, CCB, diuretics, antiplatelets, anticoagulants, antidiabetics, statin). Baseline characteristics were considered well matched between the two groups if the standardized mean differences (SMD) were ≤ 0.1.

To ensure unbiased estimation of the causal effect of SHS, we adopted the “doubly robust estimation” approach by running multivariable outcome regression after PSM as an additional adjustment, using the same covariates used in PSM in the multivariable model.^17^ To account for competing risk, the multivariable Fine–Gray regression model was selected to calculate the adjusted subdistribution hazard ratio (aSHR) and the corresponding 95% confidence intervals (CIs) to evaluate the risk of incident dementia and dementia‐related mortality in patients with and without SHS, with all‐cause death defined as the competing event. The multivariable Cox proportional hazards regression model was used to calculate the adjusted hazard ratios (aHRs) and corresponding 95% CI for the risk of all‐cause mortality. The associations between SHS and subtypes of dementia, including AD, vascular dementia, and unspecified dementia, were further calculated separately. The risk of incident dementia, dementia‐related mortality, and all‐cause mortality was also evaluated at 2‐ and 3‐year follow‐up.

Subgroup analyses were performed to evaluate the 1 year risk of incident dementia in relevant clinical subgroups using the multivariable Fine–Gray model for sex, age, baseline HTN, baseline DM, and stroke subtypes.

Two sensitivity analyses were conducted to ascertain the robustness of our findings. First, we performed conventional Cox regression analysis without considering all‐cause death as a competing risk. Second, we used the inverse probability of treatment weighting (IPTW) to further adjust for confounders. All baseline covariates of each individual were logistically regressed to calculate the propensity score of developing SHS. IPTW creates a pseudo‐population by assigning individuals with weights that correspond to the inverse of their propensity scores, such that confounders are equally distributed between patients with and without SHS.^18^ After applying IPTW, baseline characteristics were considered well balanced between the two groups if the SMDs were ≤ 0.1. Multivariable Fine–Gray and Cox regression analyses were subsequently conducted for the primary and secondary outcomes.

An exploratory analysis was performed to investigate the impact of post‐stroke management on the risk of incident dementia. In particular, physicians’ adherence to appropriate antithrombotic therapy (“A” criterion) and comorbidities optimization (“C” criterion), as outlined in the integrated ABC_stroke_ pathway in the position paper of the European Society of Cardiology (ESC) Council on Stroke,^13^ were evaluated at 30 days post‐stroke in patients with and without SHS. With reference to the position paper, the definitions adopted in this study for the “A” criterion and “C” criterion were as follows.

“A” criterion: For appropriate antithrombotic therapy, patients with AF were adherent to the “A” criterion if they had been prescribed oral anticoagulants either as warfarin or NOACs after stroke. For patients without AF, they were adherent if appropriate antiplatelet therapy, such as aspirin or a P2Y12 inhibitor, was prescribed.

“C” criterion: For comorbidities optimization, we considered the use of a statin in all patients and management of HTN, IHD, and DM if indicated. Optimal medical treatment for the listed comorbidities was defined as follows: (1) for HTN, treatment with monotherapy or combination therapy of ACEi/ARB, CCB, or diuretics; (2) for IHD, treatment with ACEi/ARB and beta‐blocker; (3) for DM, treatment with insulin or metformin. Patients were considered adherent to the “C” criterion when all comorbidities were properly treated, and a statin was prescribed.

Patients were categorized into four groups based on whether they had SHS and whether post‐stroke adherent care to the two criteria above was prescribed. The same multivariable Fine–Gray model used in the main analysis was applied to evaluate the 1 year risk of incident dementia for each group, with SHS patients who did not receive adherent care as the reference group. Cumulative incidence curves for the 1 year risk of incident dementia between patients with and without SHS, and among the four groups, were illustrated. All statistical analyses were performed using R, version 4.3.1, The R Foundation, 2023. A two‐way *P* value < 0.05 was considered statistically significant.

## 3 RESULTS

### 3.1 Study cohort

Overall, we included 130,605 patients (mean age 70.3 ± 13.3 years, 55.3% male) with first‐ever IS in this analysis (Figure S1). A total of 12,696 (9.7%) patients developed SHS within 30 days after stroke. Of these, 1121 (0.9%) developed HF, 4077 (3.1%) developed IHD, 8746 (6.7%) developed AF/AFL, and 73 (0.1%) developed VT/VF (Table 1). Prior to PSM, patients with SHS were older, more likely to be female, and less likely to be smokers or drinkers. In addition, patients with SHS were more likely to have a medical history of HTN, DM, and CKD, while less likely to have dyslipidemia or cancers than patients without SHS. Baseline medication usage was also higher in patients with SHS. A summary of the baseline characteristics is shown in Table 2. After 1:1 PSM, 12,624 patients were matched in each group, and all baseline covariates were well balanced between the two groups with SMDs of ≤ 0.1 (Table S2 in supporting information).

**TABLE 1:** Incidence of stroke–heart syndrome within 30 days post‐stroke.

	All population (*n* = 130,605)	
	Events (*n*)	Proportion (%)
Composite of stroke–heart syndrome	12,696	9.7
HF	1121	0.9
IHD	4077	3.1
AF/AFL	8746	6.7
VT/VF	73	0.1

Abbreviations: AF, atrial fibrillation; AFL, atrial flutter; HF, heart failure; IHD, ischemic heart disease; VF, ventricular fibrillation; VT, ventricular tachycardia.

**TABLE 2:** Baseline characteristics of patients with and without SHS before PSM.

	All patients (*n* = 130,605)	Without SHS (*n* = 117,909)	With SHS (*n* = 12,696)	*p* value	SMD before PSM	SMD after PSM
Age (years)	70.3 ± 13.3	69.7 ± 13.4	75.78 ± 11.3	<0.001	0.490	0.021
Male	72,181 (55.3)	65,685 (55.7)	6496 (51.2)	<0.001	0.091	0.006
Smoking	7844 (6.0)	7184 (6.1)	660 (5.2)	<0.001	0.039	0.005
Alcohol use	4408 (3.4)	4037 (3.4)	371 (2.9)	0.003	0.029	0.001
Baseline comorbidities						
Hypertension	61,882 (47.4)	55,060 (46.7)	6822 (53.7)	<0.001	0.141	0.010
Diabetes mellitus	31,134 (23.8)	27,824 (23.6)	3310 (26.1)	<0.001	0.057	0.008
Dyslipidemia	38,198 (29.2)	34,630 (29.4)	3568 (28.1)	0.003	0.028	0.001
Chronic kidney disease	3027 (2.3)	2625 (2.2)	402 (3.2)	<0.001	0.058	0.001
Chronic liver disease	3019 (2.3)	2696 (2.3)	323 (2.5)	0.071	0.017	0.018
Neoplasms	9329 (7.1)	8544 (7.2)	785 (6.2)	<0.001	0.042	0.009
Baseline medication use						
ACEi	21,309 (16.3)	18,410 (15.6)	2899 (22.8)	<0.001	0.184	0.011
ARB	5358 (4.1)	4630 (3.9)	728 (5.7)	<0.001	0.084	0.008
Beta‐blocker	26,702 (20.4)	22,021 (18.7)	4681 (36.9)	<0.001	0.415	0.002
CCB	43,199 (33.1)	38,105 (32.3)	5094 (40.1)	<0.001	0.163	0.008
Diuretics	10,793 (8.3)	9109 (7.7)	1684 (13.3)	<0.001	0.181	0.006
Aspirin	23,905 (18.3)	19,568 (16.6)	4337 (34.2)	<0.001	0.412	0.002
P2Y12 inhibitor	1199 (0.9)	991 (0.8)	208 (1.6)	<0.001	0.072	0.009
Warfarin	1062 (0.8)	659 (0.6)	403 (3.2)	<0.001	0.194	0.016
NOAC	237 (0.2)	125 (0.1)	112 (0.9)	<0.001	0.111	0.028
Insulin	4150 (3.2)	3721 (3.2)	429 (3.4)	0.182	0.013	0.011
Metformin	19,263 (14.7)	17,342 (14.7)	1921 (15.1)	0.206	0.012	0.002
Statin	24,425 (18.7)	21,112 (17.9)	3313 (26.1)	<0.001	0.199	0.006

*Note*: Values are shown as mean ± standard deviation or *n* (%).

Abbreviations: ACEi, angiotensin converting enzyme inhibitor; ARB, angiotensin receptor blocker; CCB, calcium channel blocker; NOAC, non‐vitamin K antagonist oral anticoagulants; PSM, propensity score matching; SHS, stroke–heart syndrome; SMD, standardized mean difference.

### 3.2 Outcomes and survival analysis

As shown in Table 3, a total of 778 (3.1%) patients developed incident dementia at 1 year after stroke. Of these, 418 new cases of dementia (3.3%) were recorded among patients with SHS compared to 360 cases (2.9%) among patients without SHS. In addition, the number of deaths recorded at 1 year follow‐up was 1742 (13.8%) in the SHS group and 1327 (10.5%) in the group without SHS. For dementia‐related mortality, 71 (0.6%) cases were reported in the SHS group, while 49 (0.4%) cases were reported in the group without SHS.

**TABLE 3:** Fine–Gray and Cox regression analyses for the risk of incident dementia, dementia‐related mortality, and all‐cause mortality in patients with and without SHS at 1, 2, and 3 years of follow‐up after PSM.

	Event number (%)	Unadjusted HR/SHR (95% CI)	*p* value	Adjusted HR/SHR (95% CI)	*p* value
*1 year follow‐up*					
Incident dementia^*^					
Without SHS	360 (2.9)	Ref.		Ref.	
With SHS	418 (3.3)	1.16 (1.01–1.34)	0.034	1.19 (1.03–1.37)	0.016
Dementia‐related mortality^*^					
Without SHS	49 (0.4)	Ref.		Ref.	
With SHS	71 (0.6)	1.45 (1.01–2.09)	0.045	1.53 (1.06–2.20)	0.022
All‐cause mortality					
Without SHS	1327 (10.5)	Ref.		Ref.	
With SHS	1742 (13.8)	1.34 (1.25–1.44)	<0.001	1.39 (1.30–1.50)	<0.001
*2 year follow‐up*					
Incident dementia^*^					
Without SHS	516 (4.1)	Ref.		Ref.	
With SHS	585 (4.6)	1.14 (1.01–1.28)	0.032	1.16 (1.03–1.31)	0.015
Dementia‐related mortality^*^					
Without SHS	122 (1.0)	Ref.		Ref.	
With SHS	158 (1.3)	1.30 (1.02–1.64)	0.031	1.35 (1.07–1.72)	0.012
All‐cause mortality					
Without SHS	2153 (17.1)	Ref.		Ref.	
With SHS	2737 (21.7)	1.31 (1.24–1.39)	<0.001	1.37 (1.29–1.45)	<0.001
*3 year follow‐up*					
Incident dementia^*^					
Without SHS	655 (5.2)	Ref.		Ref.	
With SHS	702 (5.6)	1.08 (0.97–1.20)	0.180	1.10 (0.98–1.22)	0.096
Dementia‐related mortality^*^					
Without SHS	200 (1.6)	Ref.		Ref.	
With SHS	265 (2.1)	1.33 (1.11–1.60)	0.002	1.37 (1.14–1.65)	0.001
All‐cause mortality					
Without SHS	2865 (22.7)	Ref.		Ref.	
With SHS	3589 (28.4)	1.30 (1.24–1.37)	<0.001	1.37 (1.30–1.44)	<0.001

Abbreviations: CI, confidence interval; HR, hazard ratio; PSM, propensity score matching; SHS, stroke–heart syndrome; SHR, subdistribution hazard ratio.

^*^ = Fine–Gray model was used to adjust for competing risk, with death being the competing event.

**FIGURE 1:**
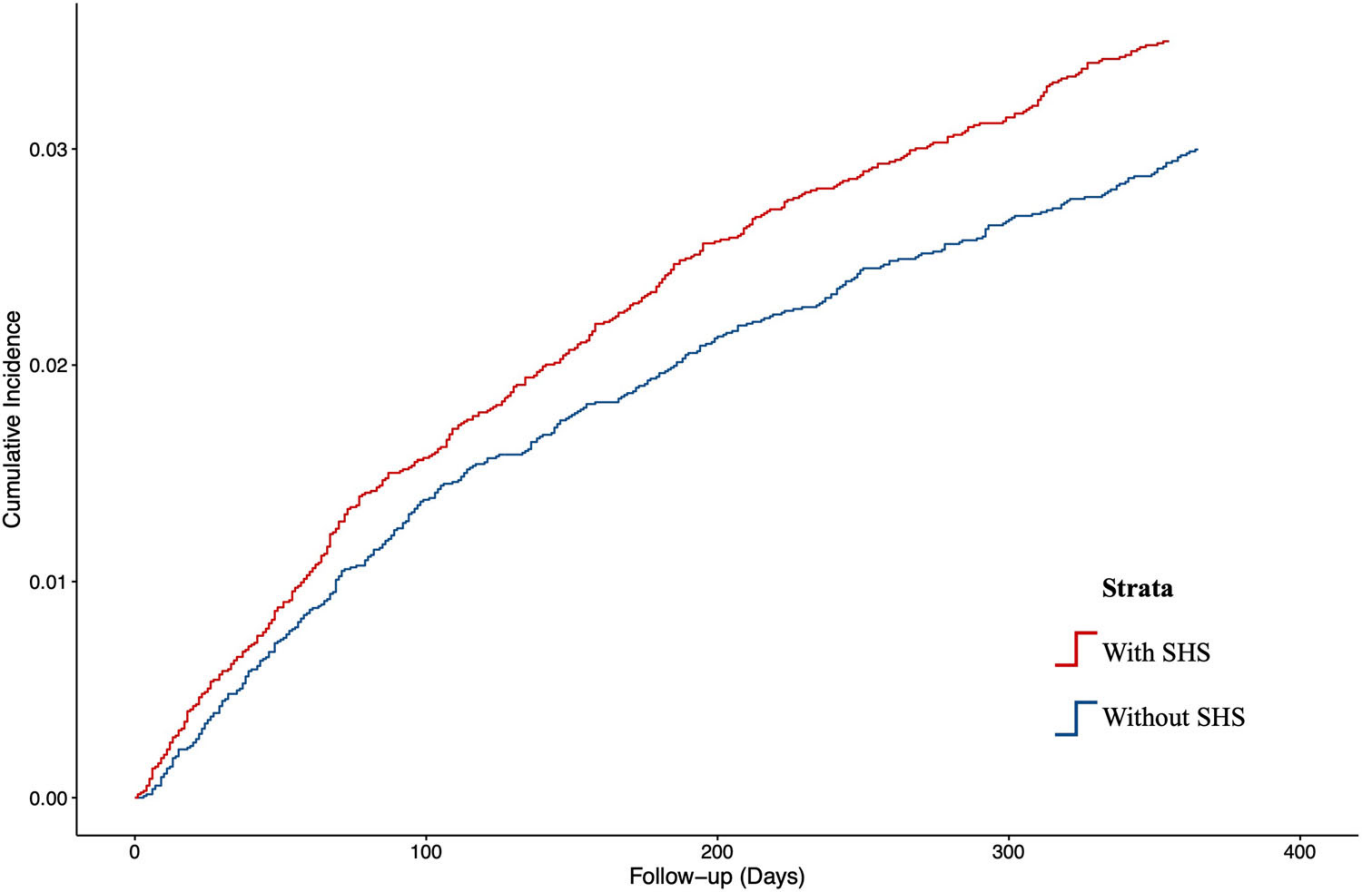
Cumulative incidence curves showing the 1 year risk of incident dementia in patients with and without SHS. SHS, stroke–heart syndrome.

At 1 year follow‐up, multivariable Fine–Gray analysis revealed that development of SHS was associated with a 19% increased risk for incident dementia (aSHR: 1.19; 95% CI: 1.03–1.37), as illustrated in the cumulative incidence curve in Figure 1. Among the various subtypes of dementia, development of SHS was associated with a significantly higher risk for vascular dementia (aSHR: 1.30; 95% CI: 1.10–1.53) but was non‐significant for AD (aSHR: 0.77; 95% CI: 0.51–1.14) and unspecified dementia (aSHR: 1.11; 95% CI: 0.74–1.68; Table 4). Compared to patients without SHS, patients with SHS were also associated with a higher risk of dementia‐related mortality (aSHR: 1.53; 95% CI: 1.06–2.20) and all‐cause mortality (aHR: 1.39; 95% CI: 1.30–1.50) at 1 year post‐stroke (Table 3, Figures S2 and S3 in supporting information).

**TABLE 4:** Fine–Gray analysis for the risk of incident dementia and its subtypes in patients with and without SHS at 1 year of follow‐up after PSM.

	Event number (%)	Unadjusted SHR (95% CI)	*p* value	Adjusted SHR (95% CI)	*p* value
All types of dementia					
Without SHS	360 (2.9)	Ref.		Ref.	
With SHS	418 (3.3)	1.16 (1.01–1.34)	0.034	1.19 (1.03–1.37)	0.016
Alzheimer's disease					
Without SHS	57 (0.5)	Ref.		Ref.	
With SHS	43 (0.3)	0.75 (0.51–1.12)	0.160	0.77 (0.51–1.14)	0.190
Vascular dementia					
Without SHS	259 (2.1)	Ref.		Ref.	
With SHS	329 (2.6)	1.27 (1.08–1.50)	0.004	1.30 (1.10‐1.53)	0.002
Unspecified dementia					
Without SHS	44 (0.3)	Ref.		Ref.	
With SHS	46 (0.4)	1.05 (0.69–1.58)	0.830	1.11 (0.74–1.68)	0.620

Abbreviations: CI, confidence interval; PSM, propensity score matching; SHR, subdistribution hazard ratio; SHS, stroke–heart syndrome.

At 2 year follow‐up, patients with SHS continued to have significantly higher risks of dementia (aSHR: 1.16; 95% CI: 1.03–1.31) compared to those without SHS (Table 3). The risk of incident dementia showed a decreasing trend as the follow‐up period extended, to which the association became non‐significant at 3‐year follow‐up (aSHR: 1.10; 95% CI: 0.98–1.22). The risk of dementia‐related mortality and all‐cause mortality were consistently elevated at 2‐ and 3‐year follow‐up (Table 3).

### 3.3 Subgroup analysis

**FIGURE 2:**
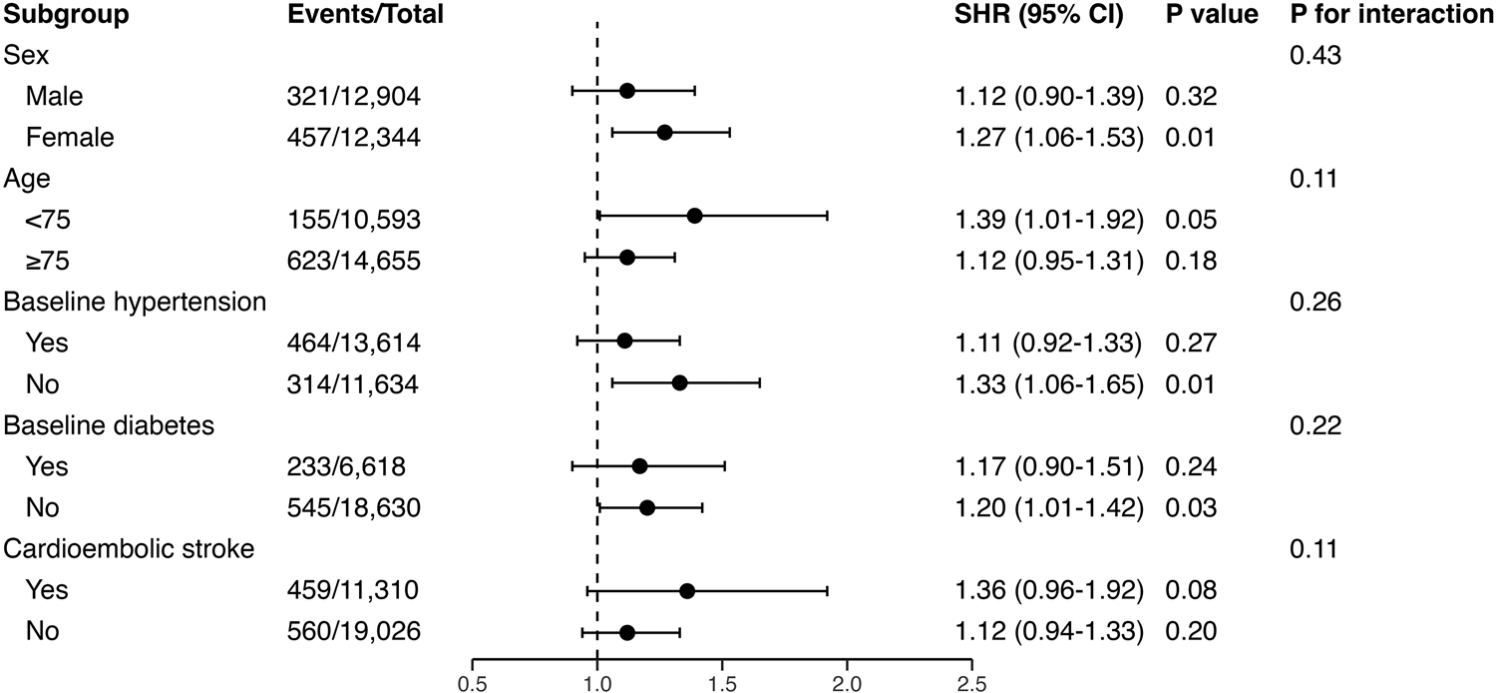
Subgroup analysis for the impact of SHS on the risk of incident dementia at 1 year of follow‐up after PSM. CI, confidence interval; PSM, propensity score matching; SHS, stroke–heart syndrome; SHR, subdistribution hazard ratio.

The results of subgroup analyses are illustrated in Figure 2. Patients with SHS were associated with a higher 1 year risk of incident dementia, except for the subgroups of male, age ≥ 75, and those with baseline HTN or DM, where this association was not observed. No statistically significant risk was found in the two separate stroke subtypes. No significant interactions across the subgroups were evident (*P* interaction all ≥ 0.05).

### 3.4 Sensitivity analysis

We performed conventional Cox regression analyses for the primary and secondary outcomes without adjusting for competing risk. In keeping with the main analysis, patients with SHS were associated with a higher 1 year risk of incident dementia (aHR: 1.23; 95% CI: 1.07–1.41) compared to patients without SHS (Table S3 in supporting information). We further adopted the Fine–Gray analysis for the clinical outcomes with IPTW as an alternative method of matching. After matching with IPTW, baseline characteristics were well balanced between the group with SHS and the group without (Table S4 in supporting information). Consistent with the main analysis, multivariable analyses after IPTW showed that patients with SHS had a higher risk of incident dementia (aSHR: 1.17; 95% CI: 1.05–1.32) at 1 year post‐stroke compared to patients with IS alone (Table S5 in supporting information).

### 3.5 Impact of post‐stroke management

**FIGURE 3:**
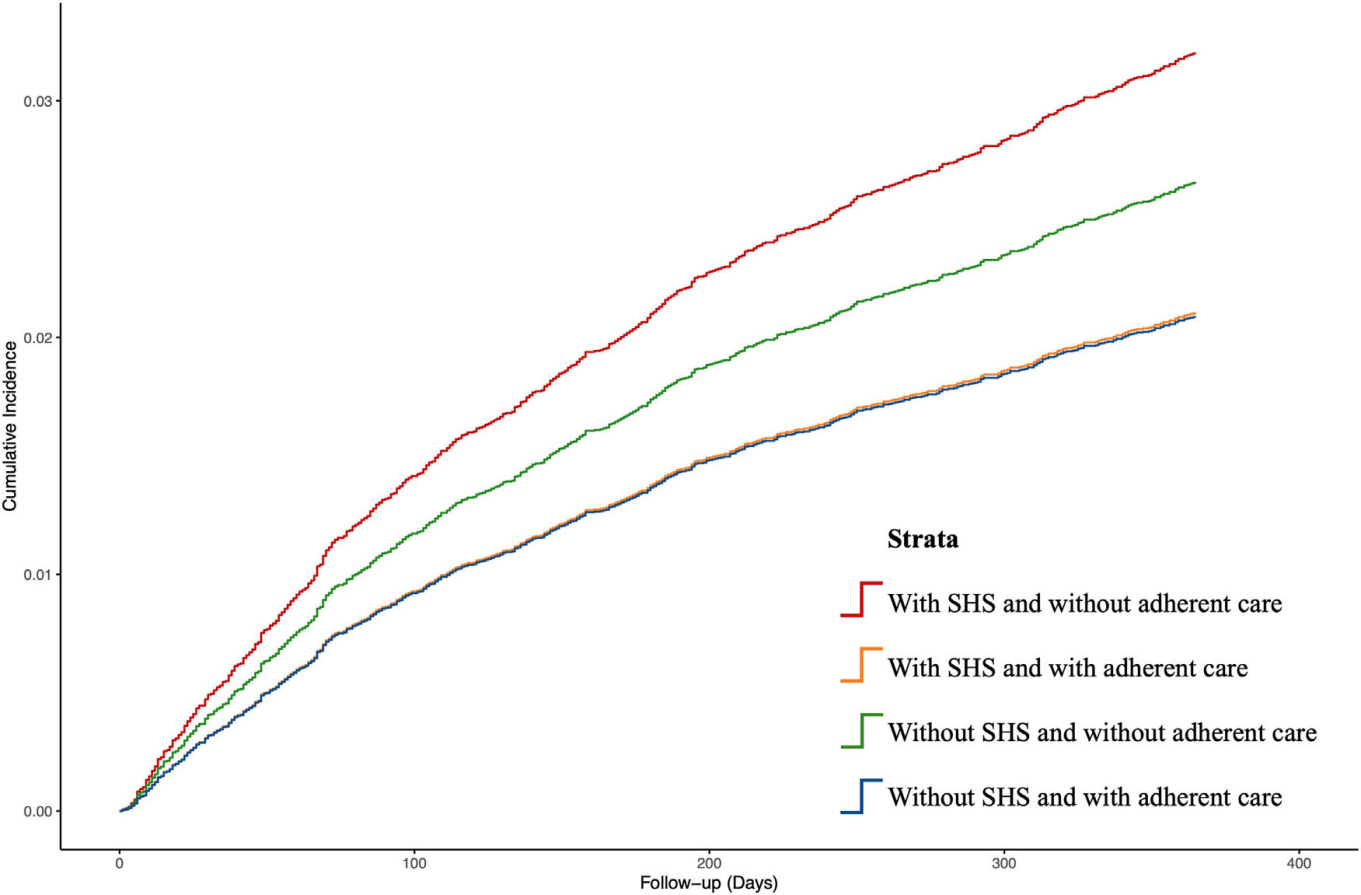
Cumulative incidence curves showing the impact of adherent care with appropriate antithrombotic therapy and comorbidities optimization on the 1 year risk of incident dementia in patients with and without SHS. SHS, stroke–heart syndrome.

At 30 days post‐stroke, adherent care according to both appropriate antithrombotic therapy (“A” criterion) and comorbidities optimization (“C” criterion) was achieved in 3407 (27.0%) patients with SHS and 5902 (46.8%) patients without SHS in the PSM cohort (Table 5). The low percentage of adherent care in patients with SHS was mainly driven by the low prescription rate of oral anticoagulants (48.9%) in patients with new‐onset AF and anti‐ischemic agents (30.8%) in patients with new‐onset IHD (Table S6 in supporting information). As for patients without SHS, the low prescription rate of statin post‐stroke (61.4%) accounted for the suboptimal percentage of adherent care in this group of patients (Table S6). Compared to SHS patients without adherent care, patients with SHS who received adherent care were associated with 32% lower 1 year risk of incident dementia (aSHR: 0.68; 95% CI: 0.53–0.89), which was lower than patients without SHS who did not receive adherent care (aSHR: 0.85, 95% CI: 0.72–1.01), and was comparable to patients without SHS who received adherent care (aSHR: 0.68, 95% CI: 0.56–0.84; Table 5). The cumulative incidence curves for these four groups of patients are illustrated in Figure 3.

**TABLE 5:** Effect of appropriate antithrombotic therapy and comorbidities optimization at 30 days post‐stroke on the risk of incident dementia at 1 year follow‐up after PSM.

	Criteria of care		
	A	C	A + C
Patients with SHS without adherent care			
*N* (%)	4990 (39.5)	7435 (58.9)	9217 (73.0)
Adjusted SHR (95% CI)	Ref.	Ref.	Ref.
Patients with SHS with adherent care			
*N* (%)	7634 (60.5)	5189 (41.1)	3407 (27.0)
Adjusted SHR (95% CI)	0.73 (0.60–0.89)	0.87 (0.71–1.07)	0.68 (0.53–0.89)
Patients without SHS without adherent care			
*N* (%)	2233 (17.7)	6043 (47.9)	6722 (53.2)
Adjusted SHR (95% CI)	0.78 (0.59–1.03)	0.85 (0.71–1.02)	0.85 (0.72–1.01)
Patients without SHS with adherent care			
*N* (%)	10,391 (82.3)	6581 (52.1)	5902 (46.8)
Adjusted SHR (95% CI)	0.71 (0.59–0.84)	0.74 (0.61–0.91)	0.68 (0.56–0.84)

Abbreviations: A, appropriate antithrombotic therapy; C, comorbidities optimization; CI, confidence interval; PSM, propensity score matching; SHS, stroke–heart syndrome; SHR, subdistribution hazard ratio.

## 4 DISCUSSION

In this population‐based, propensity score‐matched, retrospective cohort study of 130,605 patients with first‐ever IS, our main findings are as follows: (1) 9.7% of the cohort developed new‐onset cardiovascular complications within 30 days post‐stroke (i.e., SHS); (2) development of SHS associated with a 19% increased 1 year risk for incident dementia (especially vascular dementia) and a 39% increased risk for all‐cause mortality; (3) the increased risk of dementia showed a gradual reduction with each successive year of follow‐up and became non‐significant after 3 years post‐stroke; (4) optimal post‐stroke management with appropriate antithrombotic therapy and comorbidities optimization associated with a lower risk of incident dementia.

The individual contributions of IS and cardiovascular events to dementia risk are well documented.^11^, ^19^ In the population‐based Oxford Vascular Study, the prevalence of dementia in 1 year stroke survivors was brought forward by approximately 25 years in those who had severe strokes compared to age‐ and sex‐matched controls.^10^ This heightened risk of dementia has been similarly reported in patients with AF,^20^ HF,^21^ and myocardial infarction survivors.^22^ In this contemporary cohort of patients with first‐ever IS, we found that stroke survivors with early cardiovascular complications were associated with a significantly higher 1 year risk of incident dementia. Our findings emphasize the possible bidirectional relationship of the brain–heart axis and highlight the cumulative impact of these conditions. While the exact cascade of events of this brain–heart axis has yet to be elucidated, we postulate that the increased risk of dementia associated with SHS may follow the “two‐hit hypothesis.” After IS, microinfarction, excitotoxicity, oxidative stress, blood–brain barrier dysfunction, and focal neuronal atrophy may represent the “first hit” that leaves the brain in a vulnerable state.^8^, ^23^ In patients with SHS, autonomic dysregulation, coupled with systemic release of catecholamines and inflammatory cytokines, may then mount the “second hit” that impairs cerebral perfusion, hence leading to more extensive tissue damage and accelerated cognitive decline.^5^ This may explain the higher risk of dementia, particularly vascular dementia, that was observed in the first year post‐stroke. Indeed, we found that the association between SHS and dementia gradually declined and became non‐significant at 3 years post‐stroke, consistent with previous studies.^10^, ^24^, ^25^ This apparent temporal reduction in the effect of SHS may be attributable to the stabilization of the acute vascular insult and the resolution of inflammation. The development and progression of other dementia risk factors, especially in this older age group, may also account for the reduced difference between the two groups over time. However, this hypothesis has yet to be proven.^26^

Post‐stroke cardiovascular complications are not uncommon. Consistent with prior findings that reported SHS incidence of 10% to 20%,^3^, ^4^ we found that 9.7% of patients with first‐ever IS in this cohort developed SHS. In fact, complementary to previous studies, we showed that patients with SHS in this cohort were older, more likely to be female, and with more cardiac comorbidities.^27^, ^28^, ^29^ As illustrated in the subgroup analysis, females also seem to be at a higher risk of SHS‐associated dementia than males, likely attributable to known sex differences in post‐stroke stress response, autonomic function, and inflammatory response.^30^ In addition, our subgroup analysis found that those aged < 75 and those without baseline HTN or DM might be more susceptible to SHS‐associated dementia. Therefore, while these patient subgroups might be less likely to have SHS, they could be at a higher risk of SHS‐associated dementia if they ever do develop SHS. One potential explanation is that old age, hypertension, and diabetes mellitus are all well‐established risk factors of dementia.^16^ Hence, patients with these comorbidities at baseline are already at elevated risk of dementia; thus, the cumulative effect of SHS may be less prominent. Overall, the association of SHS with increased risk of incident dementia was not modified by sex, age, baseline HTN or DM, and stroke subtypes (*P* interaction all ≥ 0.05). Nevertheless, several prespecified subgroups were underpowered, so the results of the subgroup analysis should be interpreted with caution. Future prospective studies exploring the impact of patient covariates and stroke etiology on SHS‐associated dementia are warranted.

Among the various manifestations of SHS, AF/AFL is the most common cardiac complication that was reported in 6.7% of post‐stroke patients in this cohort. Although the impact of systematic AF screening is not firmly established yet, the incidence of post‐stroke AF does vary significantly depending on the method and duration of monitoring.^31^ A meta‐analysis showed that the overall AF detection yield after sequential cardiac monitoring up to the second ambulatory period was up to 24% compared to 5% with just in‐patient cardiac telemetry and in‐hospital Holter monitoring.^32^ Hence, looking harder and longer with more sophisticated methods, as advocated by the European Heart Rhythm Association (EHRA), would likely increase post‐stroke AF detection in the context of SHS.^33^

Despite recent advances in the understanding of the brain–heart axis, no specific treatment exists for preventing post‐stroke cardiovascular complications. Targeting inflammation through the inhibition of interleukin‐1 and sympathetic overactivation by blocking β‐adrenergic receptors has been proposed,^5^ but more robust evidence is warranted to support their recommendation for clinical use. Similarly, there are currently no well‐established pharmacological interventions for vascular dementia. Given the shared risk factors between IS, SHS, and dementia, optimizing these comorbidities would be fundamental in mitigating the consequences of deleterious brain–heart interactions.

In this context, this may be addressed through the ABC_stroke_ pathway, an integrated care approach to optimize the management of stroke and associated heart disease, outlined in the position paper of the ESC Council on Stroke.^13^ The ABC_stroke_ pathway is based on three pillars of management: (A) appropriate antithrombotic therapy; (B) better functional and psychological status; (C) cardiovascular risk factors and comorbidity optimization (including lifestyle changes). The effect of the ABC_stroke_ pathway had been validated in two IS cohorts in which optimal management according to the ABC_stroke_ pathway was associated with lower risks of stroke recurrence, major cardiovascular events, and mortality.^34^, ^35^ In this study, an exploratory analysis was done to evaluate the impact of appropriate antithrombotic therapy (“A” criterion) and comorbidities optimization (“C” criterion) on the risk of incident dementia. Compared to SHS patients without adherent care, patients with SHS who received adherent care were associated with a significantly lower risk of incident dementia, comparable even to patients who did not develop SHS and received adherent care. This demonstrates the potential benefits of the ABC_stroke_ pathway, beyond lowering mortality and adverse cardiovascular outcomes, in reducing the risk of cognitive impairment. However, the proportions of patients meeting both criteria in this cohort were suboptimal, and these real‐world data reveal important treatment gaps that warrant physicians’ attention to optimize patient outcomes. It should be noted that the impact of functional and psychological status (“B” criterion) was not assessed in this study, as these data were available only in a small subset of patients. However, post‐stroke depression,^16^, ^36^ physical inactivity,^37^, ^38^ and social isolation^39^, ^40^ are common in stroke survivors, and have been shown to be associated with the development of dementia.^16^ Therefore, it can be inferred that targeted interventions for the “B” criterion, including physical rehabilitation^41^ and interventions for post‐stroke depression,^42^ may also be beneficial in reducing the risk of cognitive impairment. The utility of standardized post‐stroke protocols, according to the ABC_stroke_ pathway, should be tested in clinical trials to guide best practices in patient management and enhance recovery outcomes.

We acknowledge some potential limitations of our data and its interpretation. First, due to the retrospective and observational nature of this study, causality cannot be established, and there may be residual confounding despite our use of PSM and IPTW to balance the clinically relevant variables between the two groups. For instance, data on proxies of cognitive and brain reserve, such as educational level, lifestyle factors, and premorbid cognitive function, were not available in CDARS, which may have led to bias in the results. Second, diagnoses rely on the accuracy of recording of ICD codes and may be subject to misclassification. Third, only a small proportion of patients had neuroimaging and cardiac investigations data on stroke severity, lesion characteristics, and etiology of IS, which limited our ability to explore the effect of these factors on the risk of dementia. Fourth, findings from this study are mostly restricted to Asian patients and thus future studies conducted in other ethnic groups are warranted. Fifth, treatment may have fallen short of full adherence to the ABC_stroke_ pathway in patients who were thought to have a poor prognosis, or with severe neurological deficits and disabilities after stroke, which may have resulted in a degree of selection bias. Last, the exploratory analysis on post‐stroke management was assessed within 30 days post‐stroke; thus, changes in treatment that might have happened during follow‐up would not be captured.

In conclusion, the development of SHS was associated with increased 1 year risk of dementia, dementia‐related mortality, and all‐cause mortality. Optimal post‐stroke management with appropriate antithrombotic therapy and comorbidities optimization may reduce this heightened risk of cognitive impairment.

## CONFLICT OF INTEREST STATEMENT

C.T.W.T., S.E.C., T.B., A.C.W.L., Q.W.R., J.Y.H., M.Z.W., W.L.G., R.G., J.N.Z., Y.C.N., Y.H.C., H.F.T., A.H.A.R., G.Y.H.L. report no conflicts of interest. B.J.R.B. has received research grant and consulting fees from Huawei EU and BMS/Pfizer. J.F.S. has received a grant from Deutsche Herzstiftung; consulting fees from Medtronic; honoraria from AstraZeneca and Bristol‐Myers Squibb; and reports participation in the CABA‐HF trial, all outside the submitted work. K.K.L. has received grants from University Grants Committee Hong Kong, World Stroke Organisation, Hong Kong Government Food & Health Bureau, and Pfizer; consulting fees from Amgen, Boehringer Ingelheim, Daiichi Sankyo, and Sanofi; honoraria from Amgen and Boehringer Ingelheim; support for attending meetings from Pfizer and Daiichi Sankyo; and reports a role as Honorary Secretary, Hong Kong Stroke Society, all outside the submitted work. K.H.Y. has received grants from the National Natural Science Foundation of China (No. 82270400), the Natural Science Foundation of Guangdong Province (No. 2023A1515010731), and Sanming Project of Medicine in Shenzhen (No. SZSM202411021). Author disclosures are available in the supporting information.

## CONSENT STATEMENT

As patient data were de‐identified in CDARS, the need for individual consent was waived. The study has been approved by the institutional review board of the University of Hong Kong/ Hospital Authority Hong Kong West Cluster (IRB Reference Number: UW 24‐187).

## Supporting information



Supporting Information

Supporting Information
