# Supplementary material for: Stroke–heart syndrome and risk of incident dementia among patients with first‐ever ischemic stroke: A territory‐wide population‐based cohort study
Source: Alzheimers Dement. 2025 Sep 18;21(9):e70716. doi: 10.1002/alz.70716 (PMC12444938; doi:10.1002/alz.70716)

**SUPPLEMENTARY MATERIALS**

**Table S1**. ICD-9-CM codes used for the definition of covariates

| **Covariates** | **ICD-9-CM codes** |
| --- | --- |
| Ischaemic stroke | 433, 434, 436 |
| Cardioembolic stroke | 434.10, 434.11 |
| Dementia | 290, 294.1, 331.0 |
| Alzheimer’s disease | 331.0 |
| Vascular dementia | 290.40, 290.41, 290.42, 290.43 |
| Unspecified dementia | 290.0, 290.10, 290.11, 290.12, 290.13, 290.20, 290.21, 290.3, 290.9, 294.1 |
| Heart failure | 398.91, 402.01, 402.11, 402.91, 404.01, 404.03, 404.11, 404.13, 404.91, 404.93, 428 |
| Ischaemic heart disease | 410-414 |
| Atrial fibrillation and flutter | 427.3 |
| Ventricular tachycardia/fibrillation | 427.1, 427.4 |
| Hypertension | 401-405, 437.2 |
| Diabetes mellitus | 250 |
| Dyslipidaemia | 272, 272.1, 272.2, 272.3, 272.4 |
| Chronic kidney disease | 585 |
| Chronic liver disease | 571-573 |
| Neoplasms | 140-239 |
| Smoking | 491, 492, 496 |
| Alcohol use | 291, 303, 305.0, 571.0, 571.1, 571.2, 571.3, E980.8, E980.9 |

ICD-9-CM = International Classification of Diseases, Ninth Revision, Clinical Modification.

**Table S2.** Baseline characteristics of patients with and without SHS after PSM

|  | **Without SHS**  **(n=12,624)** | **With SHS**  **(n=12,624)** | **SMD after PSM** |
| --- | --- | --- | --- |
| Age (years) | 76.0 ± 11.5 | 75.8 ± 11.3 | 0.021 |
| Male | 6,434 (51.0) | 6,470 (51.3) | 0.006 |
| Smoking | 643 (5.1) | 658 (5.2) | 0.005 |
| Alcohol use | 371 (2.9) | 369 (2.9) | 0.001 |
| Baseline comorbidities |  |  |  |
| Hypertension | 6,839 (54.2) | 6,775 (53.7) | 0.010 |
| Diabetes mellitus | 3,330 (26.4) | 3,288 (26.0) | 0.008 |
| Dyslipidaemia | 3,537 (28.0) | 3,544 (28.1) | 0.001 |
| Chronic kidney disease | 401 (3.2) | 399 (3.2) | 0.001 |
| Chronic liver disease | 357 (2.8) | 320 (2.5) | 0.018 |
| Neoplasms | 754 (6.0) | 782 (6.2) | 0.009 |
| Baseline medication use |  |  |  |
| ACEi | 2,921 (23.1) | 2,864 (22.7) | 0.011 |
| ARB | 696 (5.5) | 719 (5.7) | 0.008 |
| Beta-blocker | 4,608 (36.5) | 4,619 (36.6) | 0.002 |
| CCB | 5,113 (40.5) | 5,062 (40.1) | 0.008 |
| Diuretics | 1,628 (12.9) | 1,655 (13.1) | 0.006 |
| Aspirin | 4,316 (34.2) | 4,304 (34.1) | 0.002 |
| P2Y12 inhibitor | 192 (1.5) | 206 (1.6) | 0.009 |
| Warfarin | 319 (2.5) | 352 (2.8) | 0.016 |
| NOAC | 66 (0.5) | 94 (0.7) | 0.028 |
| Insulin | 452 (3.6) | 426 (3.4) | 0.011 |
| Metformin | 1,916 (15.2) | 1,908 (15.1) | 0.002 |
| Statin | 3,313 (26.2) | 3,277 (26.0) | 0.006 |

Values are shown as mean ± standard deviation or n (%).

SHS = Stroke-Heart Syndrome; PSM = propensity score matching; SMD = standardised mean difference; ACEi = angiotensin converting enzyme inhibitor; ARB = angiotensin receptor blocker; CCB = calcium channel blocker; NOAC = non-vitamin K antagonist oral anticoagulants.

**Table S3.** Cox regression analysis without competing risk for the risk of incident dementia, dementia-related mortality and all-cause mortality in patients with and without SHS at 1 year of follow-up after PSM

|  | **Unadjusted**  **HR**  **(95% CI)** | ***P***  **value** | **Adjusted**  **HR**  **(95% CI)** | ***P***  **value** |
| --- | --- | --- | --- | --- |
| **Incident dementia** |  |  |  |  |
| Without SHS | Ref. |  | Ref. |  |
| With SHS | 1.19 (1.03-1.37) | 0.017 | 1.23 (1.07-1.41) | 0.005 |
| **Dementia-related mortality** |  |  |  |  |
| Without SHS | Ref. |  | Ref. |  |
| With SHS | 1.49 (1.03-2.14) | 0.033 | 1.60 (1.11-2.31) | 0.011 |
| **All-cause mortality** |  |  |  |  |
| Without SHS | Ref. |  | Ref. |  |
| With SHS | 1.34 (1.25-1.44) | <0.001 | 1.39 (1.30-1.50) | <0.001 |

SHS = Stroke-Heart Syndrome; PSM = propensity score matching; HR = hazard ratio; hazard ratio; CI = confidence interval.

**Table S4**. Baseline characteristics of patients with and without SHS after IPTW

|  | **Without SHS**  **(n=** **130,654)** | **With SHS**  **(n=** **129,136)** | **SMD after IPTW** |
| --- | --- | --- | --- |
| Age (years) | 70.3 ± 13.4 | 71.0 ± 12.8 | 0.048 |
| Male | 72,207.4 (55.3) | 72,739.4 (56.3) | 0.021 |
| Smoking | 7,846.6 (6.0) | 7,775.1 (6.0) | 0.001 |
| Alcohol use | 4,412.0 (3.4) | 4,489.7 (3.5) | 0.005 |
| Baseline comorbidities |  |  |  |
| Hypertension | 68,782.3 (52.6) | 68,423.8 (53.0) | 0.007 |
| Diabetes mellitus | 99,522.7 (76.2) | 98,361.2 (76.2) | <0.001 |
| Dyslipidaemia | 38,163.5 (29.2) | 37,428.7 (29.0) | 0.005 |
| Chronic kidney disease | 3,035.5 (2.3) | 3,491.5 (2.7) | 0.024 |
| Chronic liver disease | 3,024.8 (2.3) | 3,135.8 (2.4) | 0.007 |
| Neoplasms | 68,782.3 (52.6) | 68,423.8 (53.0) | 0.004 |
| Baseline medication use |  |  |  |
| ACEi | 21,312.4 (16.3) | 20,300.4 (15.7) | 0.016 |
| ARB | 5,373.7 (4.1) | 5,373.4 (4.2) | 0.002 |
| Beta-blocker | 26,732.5 (20.5) | 26,238.4 (20.3) | 0.004 |
| CCB | 43,218.9 (33.1) | 41,883.0 (32.4) | 0.014 |
| Diuretics | 10,805.5 (8.3) | 10,595.3 (8.2) | 0.002 |
| Aspirin | 24,463.5 (18.7) | 23,953.8 (18.5) | 0.004 |
| P2Y12 inhibitor | 20,987.3 (16.1) | 19,968.5 (15.5) | 0.016 |
| Warfarin | 1,335.5 (1.0) | 1,322.5 (1.0) | <0.001 |
| NOAC | 24,424.4 (18.7) | 23,952.6 (18.5) | 0.004 |
| Insulin | 21,312.4 (16.3) | 20,300.4 (15.7) | 0.016 |
| Metformin | 5,373.7 (4.1) | 5,373.4 (4.2) | 0.002 |
| Statin | 26,732.5 (20.5) | 26,238.4 (20.3) | 0.004 |

Values are shown as mean ± standard deviation or n (%).

SHS = Stroke-Heart Syndrome; IPTW = inverse probability of treatment weighting; SMD = standardised mean difference; ACEi = angiotensin converting enzyme inhibitor; ARB = angiotensin receptor blocker; CCB = calcium channel blocker; NOAC = non-vitamin K antagonist oral anticoagulants.

**Table S5.** Fine-Gray and Cox regression analyses for the risk of incident dementia, dementia-related mortality and all-cause mortality in patients with and without SHS at 1 year of follow-up after IPTW

|  | **Unadjusted**  **HR/SHR**  **(95% CI)** | ***P***  **value** | **Adjusted**  **HR/SHR**  **(95% CI)** | ***P***  **value** |
| --- | --- | --- | --- | --- |
| **Incident dementia**^*^ |  |  |  |  |
| Without SHS | Ref. |  | Ref. |  |
| With SHS | 1.19 (1.06-1.34) | 0.003 | 1.17 (1.05-1.32) | 0.006 |
| **Dementia-related mortality**^*^ |  |  |  |  |
| Without SHS | Ref. |  | Ref. |  |
| With SHS | 1.26 (0.95-1.68) | >0.050 | 1.26 (0.95-1.67) | >0.050 |
| **All-cause mortality** |  |  |  |  |
| Without SHS | Ref. |  | Ref. |  |
| With SHS | 1.80 (1.71-1.89) | <0.001 | 1.35 (1.30-1.45) | <0.001 |

SHS = Stroke-Heart Syndrome; IPTW = inverse probability of treatment weighting; HR = hazard ratio; SHR = subdistribution hazard ratio; CI = confidence interval.

* = Fine-Gray model was used to adjust for competing risk, with death being the competing event.

**Table S6.** Diagnoses and medications prescribed in patients with and without SHS at 30-days post-stroke

| **Patients with SHS (n=12,624)** | **Proportion (%)** |
| --- | --- |
| Post-stroke AF | 8,685/12,624 (68.8) |
| OAC prescribed | 4,250/8,685 (48.9) |
| Antiplatelet prescribed | 8,497/12,624 (67.3) |
| Statin prescribed | 8,098/12,624 (64.1) |
| Hypertension | 6,775/12,624 (53.7) |
| Ischaemic heart disease | 4,061/12,624 (32.2) |
| Diabetes mellitus | 3,288/12,624 (26.0) |
| Anti-hypertensives prescribed | 5,168/6,775 (76.3) |
| Anti-ischaemic agents prescribed | 1,250/4,061 (30.8) |
| Anti-diabetics prescribed | 2,066/3,288 (62.8) |
| **Patients without SHS (n=12,624)** |  |
| Antiplatelet prescribed | 10,391/12,624 (82.3) |
| Statin prescribed | 7,749/12,624 (61.4) |
| Hypertension | 6,839/12,624 (54.2) |
| Diabetes mellitus | 3,330/12,624 (26.4) |
| Anti-hypertensives prescribed | 5,413/6,839 (79.1) |
| Anti-diabetics prescribed | 2,062/3,330 (61.9) |

SHS = Stroke-Heart Syndrome; AF = atrial fibrillation; OAC = oral anticoagulants.

**Figure S1.** Flow chart of the study cohort


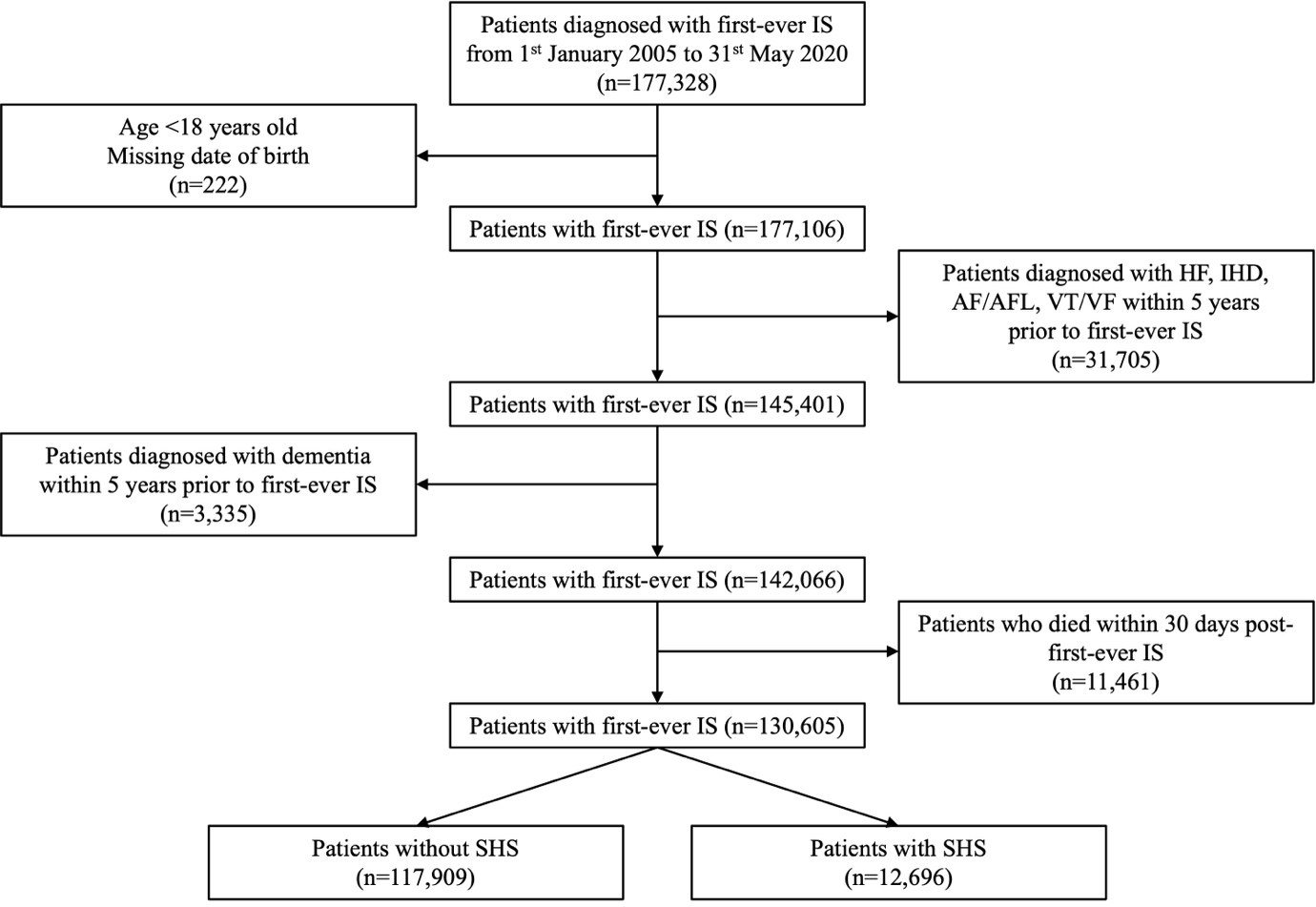


IS = ischaemic stroke; HF = heart failure; IHD = ischaemic heart disease; AF = atrial fibrillation; AFL = atrial flutter; VT = ventricular tachycardia; VF = ventricular fibrillation; SHS = Stroke-Heart Syndrome.

**Figure S2.** Cumulative incidence curves showing the 1-year risk of dementia-related mortality in patients with and without Stroke-Heart Syndrome (SHS)


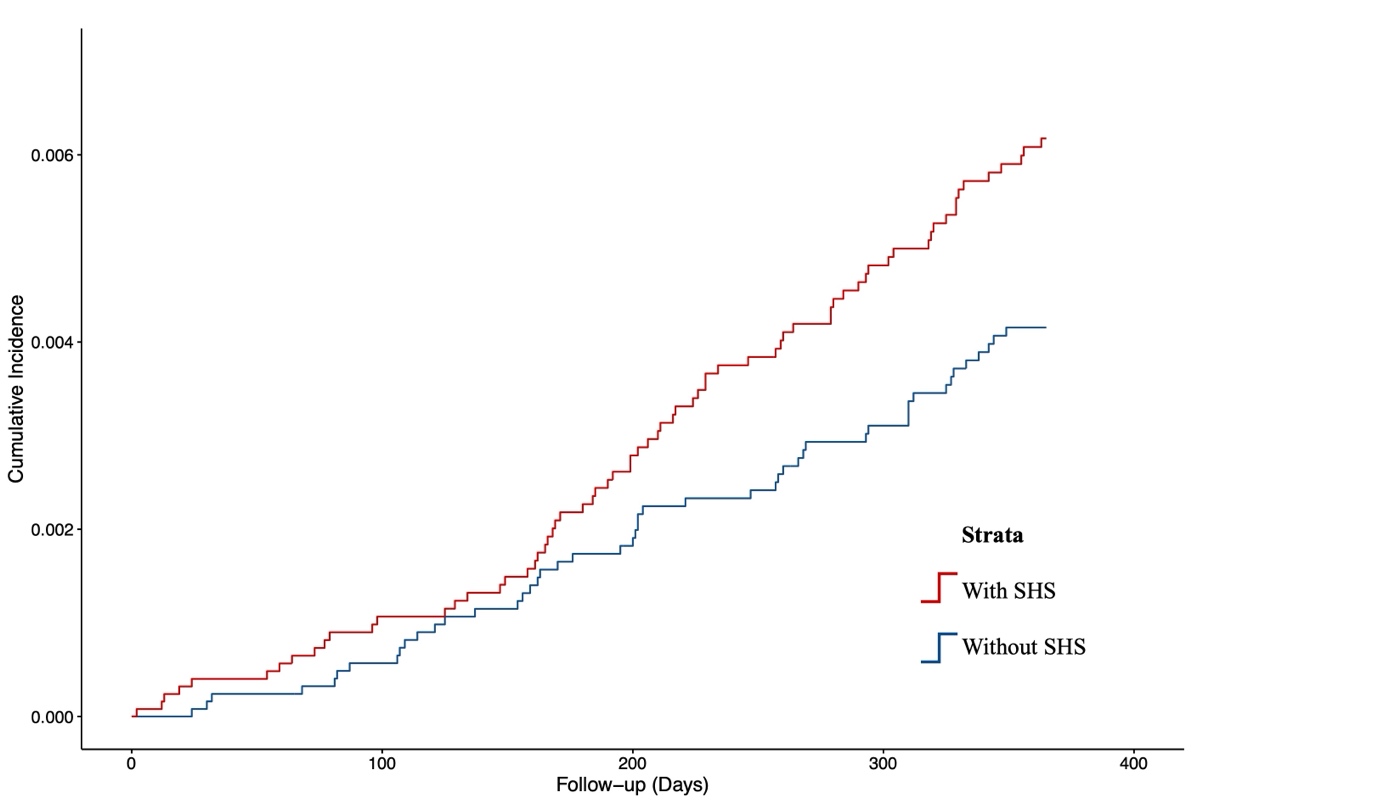


**Figure S3.** Cumulative incidence curve showing the 1-year risk of all-cause mortality in patients with and without Stroke-Heart Syndrome (SHS)


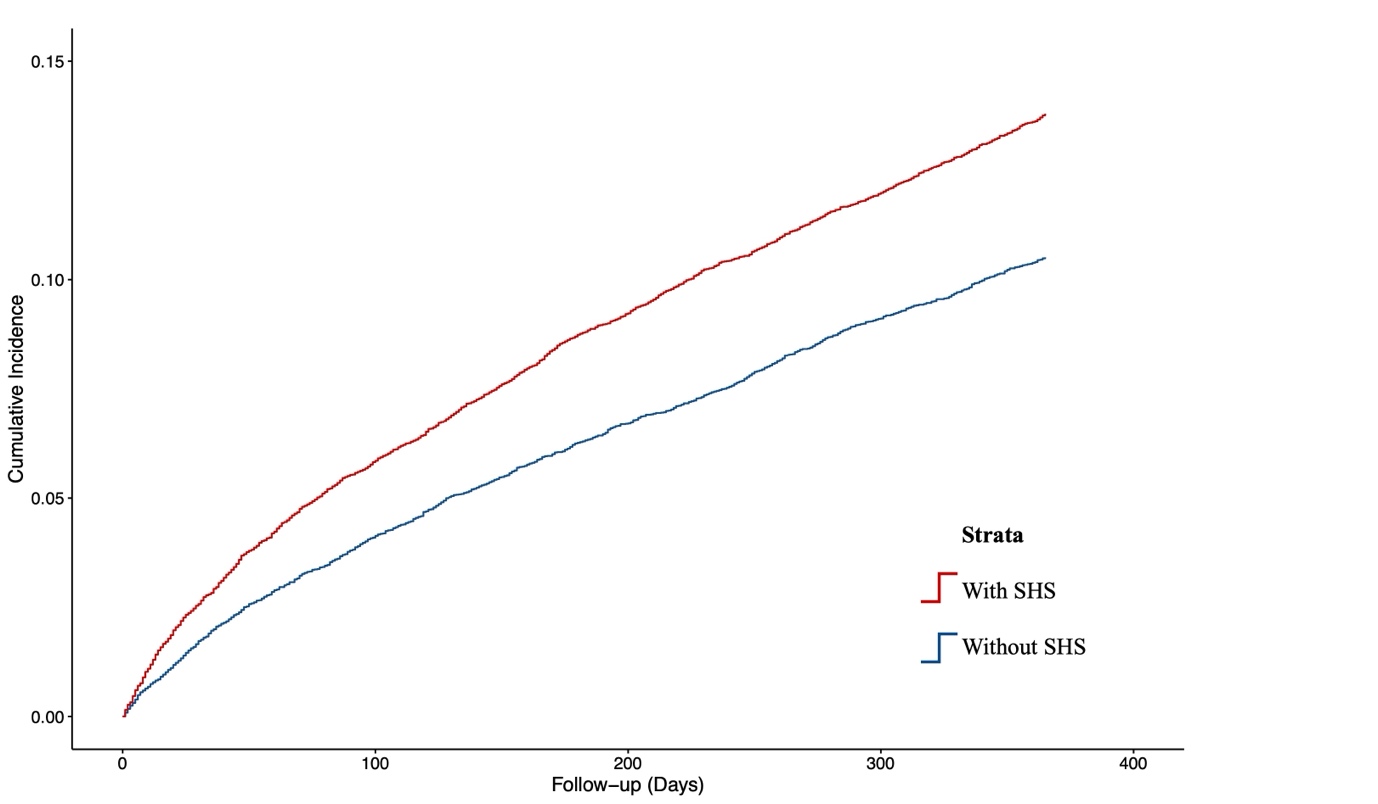

Supplement: Supplementary file 2 — Supporting Information [file ALZ-21-e70716-s001.docx]
